# The role of microglia in Alzheimer's disease and progress of treatment

**DOI:** 10.1002/ibra.12023

**Published:** 2022-02-22

**Authors:** Yi‐Huan Guan, Ling‐Jing Zhang, Shi‐Ya Wang, Ya‐Dan Deng, Hong‐Su Zhou, Dong‐Qing Chen, Lan‐Chun Zhang

**Affiliations:** ^1^ Department of Anesthesiology Zunyi Medical University Zunyi Guizhou China; ^2^ Department of Anesthesia Graduate School of Zunyi Medical University Zunyi Guizhou China; ^3^ Department of Zoology Kunming Medical University Kunming China

**Keywords:** aging, Alzheimer disease, growth and development, microglia

## Abstract

Microglia are permanent immune cells of the central nervous system. Microglia play an important role in the pathological process of Alzheimer's disease (AD). They are mainly involved in the uptake and clearance of amyloid‐β (Aβ), as well as the formation of neuroinflammation. We found that overactivated microglia increase Aβ and Tau, and Aβ and Tau in turn act as activators of microglia. Additionally, various cytokines and proteins, high cholesterol, and telomere shortening are all associated with microglia activation. More activated microglia induce the release of inflammatory and anti‐inflammatory factors to regulate inflammation, while microglia express multiple homologous receptors that bind to neuroimmunomodulators to prevent microglia overactivation. Moreover, aging of the body promotes neuroinflammation by increasing the response to IFN‐γ (interferon‐γ), and aging of the microglia themselves promotes AD by inducing the accumulation of large amounts of iron and reducing autophagy by regulating protein levels. Cognitive dysfunction occurs when activated microglia induce an increase in beta oligomers, promoting the production of pro‐inflammatory factors that alter the shape, composition, and density of synapses. Based on their correlation, microglia‐mediated AD therapy as well as the corresponding targets and drugs are discussed. In contrast to similar reviews, this article also summarizes some novel microglia‐mediated AD treatment methods over the recent years.

## Graphical abstract

Microglia are permanent immune cells of the central nervous system. Microglia play an important role in the pathological process of Alzheimer's disease (AD). They are mainly involved in the uptake and clearance of amyloid‐β (Aβ), as well as development of neuroinflammation. We found that overactivated microglia induce an increase in Aβ and Tau, and Aβ and Tau in turn act as activators of microglia. Additionally, various cytokines and proteins, high cholesterol, and telomere shortening are all associated with microglia activation. More activated microglia induce the release of inflammatory and anti‐inflammatory factors to regulate inflammation, while microglia express multiple homologous receptors that bind to neuroimmunomodulators to prevent microglia overactivation. Moreover, aging of the body promotes neuroinflammation by increasing the response to IFN‐γ (interferon‐γ), and aging of the microglia themselves promotes AD by inducing the accumulation of large amounts of iron and reducing autophagy by regulating protein levels. Cognitive dysfunction occurs when activated microglia induce an increase in beta oligomers, promoting the production of pro‐inflammatory factors that alter the shape, composition, and density of synapses. Based on their correlation, microglia‐mediated AD therapy as well as the corresponding targets and drugs are discussed. In contrast to similar reviews, this article also summarizes some novel microglia‐mediated AD treatment methods over the recent years.

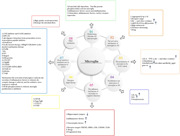

## 1 INTRODUCTION

### 1.1 Alzheimer disease

**Figure 1:**
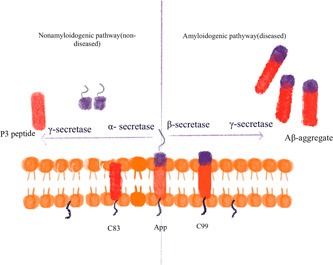
Production of Abeta (Aβ) protein [Color figure can be viewed at wileyonlinelibrary.com]

Alzheimer's disease (AD), also known as senile dementia, is a typical neurodegenerative disease, mainly manifested as progressive memory impairment, cognitive dysfunction, language impairment, and personality changes and other neuropsychiatric symptoms, and adversely affects patients' social, professional, and daily lives. With the aging of the population, about one‐third of the elderly over the age of 85 years will suffer from AD, which will place a burden on the society and families.^1^ AD is characterized by the deposition of β‐amyloid peptide (Abeta, Aβ) and Tau protein, and the progressive loss of neurons that causes dementia. Due to the deposition of mutant amyloid precursor protein (APP) and progeria protein‐1 proteins in the cortex and the hippocampus, Aβ is deposited inside the cell, leading to the formation of plaques, and outside the cell, leading to the formation of senile plaques.^2^ APP consists of 770 amino acids. Under normal shearing of APP, α‐secretase cleaves the large soluble outer domain of APPsα and secretes it into the medium. The C‐terminal fragment C83 remains in the membrane, and residue 711 is further cleaved by γ‐secretase to release the soluble P3 peptide. In the disease state, abnormal cleavage is performed by the β‐secretase of APPsβ, and the C‐terminal fragment C99 is retained in the membrane. Finally, γ‐secretase further cleaves C99 to release the insoluble Aβ peptide^3^ (Figure 1). The Tau protein accumulates and phosphorylates intracellularly to form neurofibrillary tangles, which leads to neuroinflammation.^2^, ^4^, ^5^, ^6^ The Tau protein itself has a microtubule‐binding domain, and it assembles with tubulin to form mature and stable microtubules. When the environment is rich in Aβ, the Tau protein will be hyperphosphorylated when it comes into contact with the released kinases and cytokines, causing the tubule subunits to dissociate and divide, transform into large pieces of Tau filaments and further aggregate, and finally form neural tangles. Among them, glycogen synthase kinase 3 (GSK3β) and cyclin‐dependent kinase 5 (CDK5) are the main kinases that induce phosphorylation of the Tau protein, and these kinases are activated by extracellular Aβ protein.^5^ Therefore, in recent years, increasingly more treatment options for AD have been proposed to reduce, stop, or reverse pathological phenomena by inhibiting the activation of microglia and the release of inflammatory cytokines.

### 1.2 Relationship between AD and microglia from an immune perspective

**Figure 2:**
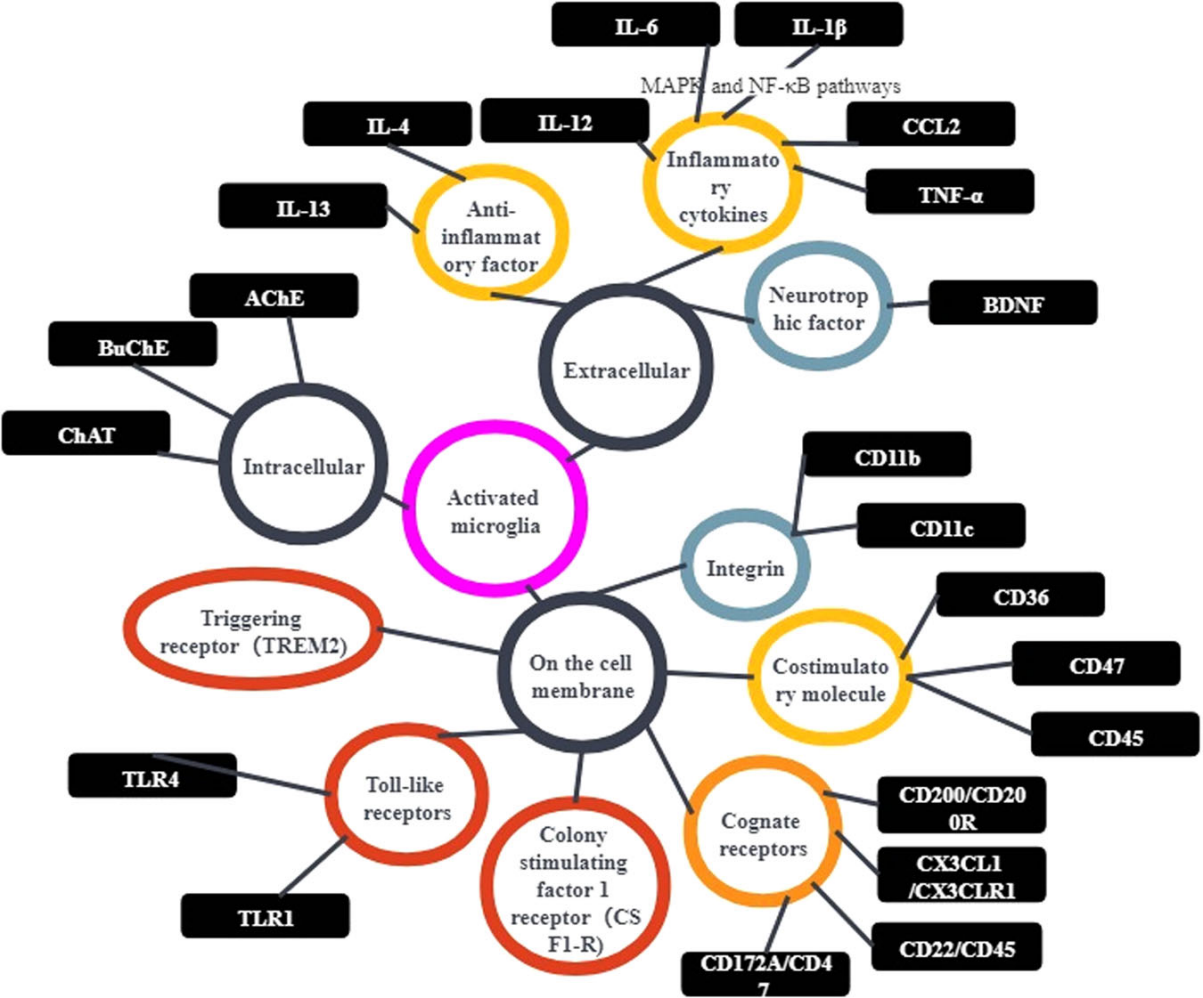
After activation of microglia, intracellular enzymes related to acetylcholine levels were changed, extracellular inflammation‐related factors, chemokines, and neurotrophic factors were increased, and the expression of inflammatory receptors on the membrane was upregulated [Color figure can be viewed at wileyonlinelibrary.com]

Microglia originate from the yolk sac. The activities of transcription factor PU.1 and interferon regulatory factor 8 (IRF8) (1–4) determine their differentiation, and they rely on Colony stimulating factor 1 (CSF1) receptor signaling for survival. Microglia play a vital role in the development of the central nervous system and in the regulation of advanced cognitive functions of the human brain. In addition, microglia can maintain the balance of the microenvironment in the body by phagocytosing dead cell, cell debris, and misfolded proteins.^7^ Microglia are roughly divided into two types with contrasting functions: M1 and M2. M1 mainly induces nitric oxide synthase 2 (iNOS/NOS2) by upregulating interferon‐γ and bacterial cell wall components, thereby producing inflammatory cytokines and nitric oxide. On the contrary, M2 releases anti‐inflammatory factors such as IL‐4 and IL‐13 to alleviate the neuro‐inflammatory response and plays a general role in neuroprotection. M1 microglia secrete various chemokines and inflammatory factors (IL‐12, IL‐6, IL‐1β, CCL2, TNF‐α) via the mitogen‐activated protein kinase (MAPK) and nuclear factor κB (NF‐κB) pathways, and also express costimulatory molecules (CD36, CD47, CD45), integrins (CD11b, CD11c), Major Histocompatibility Complex Ⅱ (MHCⅡ), and Fc receptors. On the contrary, M2 microglia produce a variety of anti‐inflammatory factors, growth factors, and neurotrophic factors (glial cell‐derived neurotrophic factor [GDNF], brain‐derived neurotrophic factor [BDNF]). These secreted products play an important role in the neurodegeneration of AD^7^ (Figure 2). In AD pathology, AD is characterized by Aβ deposition and Tau‐like protein phosphorylation, which leads to the formation of neurofibrillary tangles and neuroinflammation.^2^, ^4^, ^6^ Genome‐wide association studies support the hypothesis that immune activation response promotes the onset of AD. Several immune‐related gene variants such as Trem2 and Cd3 have been identified by this study. These genes determine the risk of AD. Moreover, Marlatt et al. used three immunohistochemical methods to study the relationship between microglia proliferation and amyloidosis, and it was found that there are a large number of proliferating microglia co‐labeled with Iba1+ in the hippocampus, especially in Aβ. The plaque area indicates that microglia are involved in the proliferation of AD and the clearance of Aβ, which may lead to neuroinflammation.^8^ Under physiological conditions, many immune cells reside in the central nervous system, activate and proliferate under the stimulation of immune‐inflammatory factors, and play a role in maintaining brain homeostasis. In AD, microglia are the main active cells, showing high differentiation and proliferation. In the process of neurodegeneration and aging, microglia acquire a unique activation phenotype, and the number of synapses decreases, leading to cognitive dysfunction. In addition, the activation of microglia induces the release of inflammatory factors such as interleukin‐1b(IL‐1b), interleukin‐(IL‐6), and tumor necrosis factor a (TNF‐a), leading to neuroinflammation. TREM2 (the trigger receptor expressed on myeloid cells 2) is a cell surface receptor selectively expressed in myeloid cells. Its genetic variation markedly increases the risk of AD, implying microglia and the innate immune system. It is a key factor in the onset of AD.^9^ Our aim is to better elucidate the mediating role of microglia in the treatment of AD by exploring the mechanism of microglia in AD, and to summarize relevant targets and drugs.

## 2 AD AND MICROGLIA

### 2.1 The mechanism of microglia activation in AD

**Figure 3:**
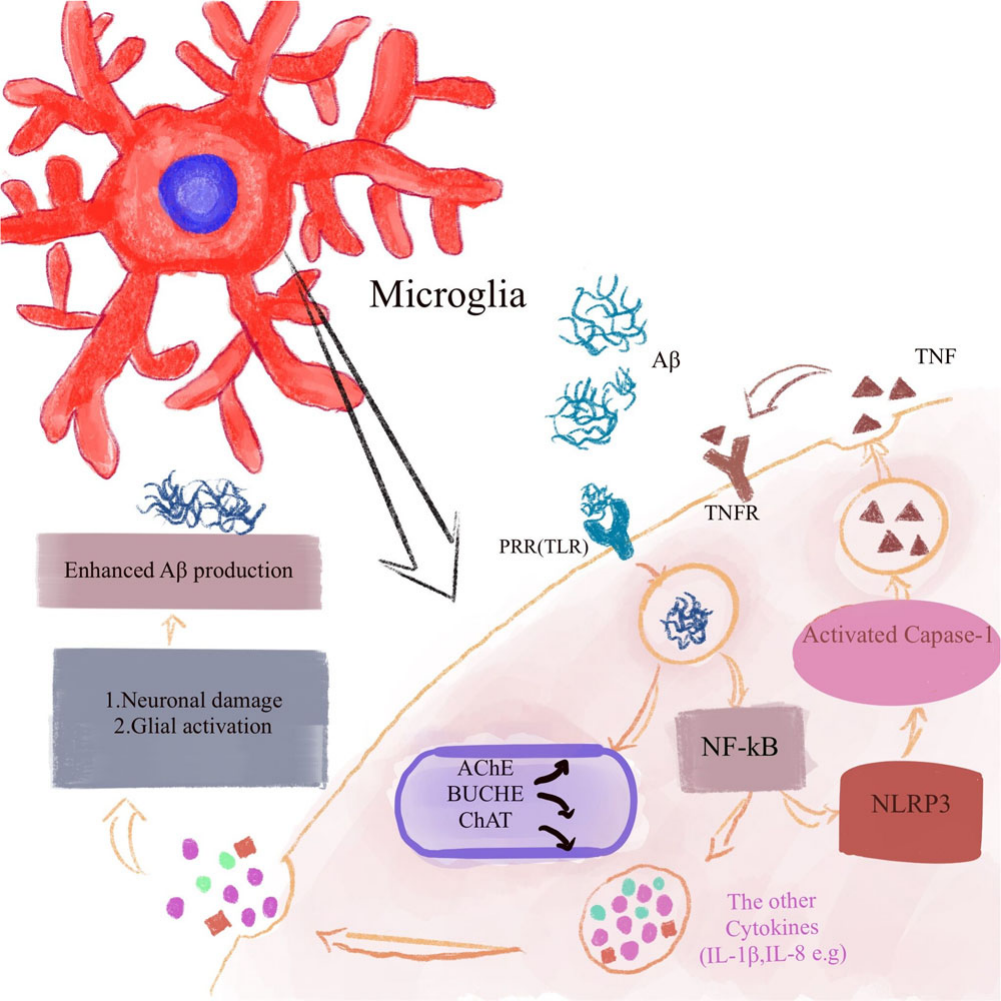
The deposition of Abeta (Aβ) binds to TLP and enters the cell through endocytosis, which leads to the activation of the inflammatory bodies (NLRP3) and the release of a large number of inflammatory factors through the NF‐KB pathway. When NLRP3 is activated, CAPase‐1, which acts as its downstream pathway, is also activated, inducing the release of large amounts of tumor necrosis factor (TNF), which in turn interacts with and activates microglia. A large number of inflammatory cytokine release leads to neuronal damage and promotes the production of Aβ [Color figure can be viewed at wileyonlinelibrary.com]

Aβ and Tau can act as activators of microglia. In the vicinity of Aβ deposition, microglia aggregate and differentiate into different activated phenotypes, the expression of various inflammatory molecules is upregulated, and CD11b and TREM2 are strongly expressed around the synapse. The transfer of Tau‐like proteins can occur through the synapses of microglia, and can also be transmitted through the phagocytosis and secretion of microglia. Toll‐like receptors (TLR4, TLR1, etc.) are also closely involved in the activation of microglia. The co‐receptors of TLR are CD14 and NOD‐like receptors such as the NLRP3 inflammasome, which are PRRs with obvious characteristics in AD models.^7^ In addition, some other studies have also confirmed these findings. Aggregated β‐ap 1‐42 activates microglia, causes changes in microglia branches, increases the proliferation index, and induces the release of TNF, while β‐ap 25‐35 does not activate microglia, and is in an amorphous state. Both peptides did not induce the release of nitric oxide (NO), but their toxicity was tested. Some studies have found that certain cytokines are related to the activation of microglia. For example, TNF, which was mentioned above, also acts on microglia in reverse and promotes microglia activation.^10^ Moreover, ifn‐γ can be upregulated to enhance the classic activation of microglia.^11^ Upregulation of the expression of proteins such as Aβ25‐35, chromogranin A (CGA), and Aβf also promotes the activation of microglia, which was demonstrated by Malmsten, Park, and Wu et al. The increase in Aβf also affects the levels of AChE, BuChE, and ChAT, and results in a significant increase in the activity of AChE (Figure 3).^12^, ^13^, ^14^ Interestingly, it was found that in AD, higher CSF PGRN is associated with more advanced disease stages and cognitive impairment. The increase in CSF PGRN is related to the increase in CSF soluble TREM2 (triggering receptor 2 on myeloid cells). Although CSF PGRN is not a diagnostic biomarker for AD, it may reflect the activation of microglia during the disease together with sTREM2.^15^ In addition, the presence of some related targets in cells can also mediate the activation of microglia, and the specific upregulation of CD200 inhibits the activation and secretion of microglia. Yasuno et al.^16^ performed PET scans on some MCI patients, AD patients, and normal individuals, and found that the wide area of DAA1106 binding to PBR was significantly increased in MCI patients, finally indicating that the activation of microglia may occur before the onset of dementia. Moreover, studies have found that the scale of the effect measured by PAM is huge, and the mediation model also promotes the activation of microglia through the accumulation of the Tau protein. Moreover, a variant (rs2997325) was found, which is a common type of variant that affects PAM and also affects the activation of microglia in the body.^17^ Most importantly, in AD, the activation of microglia has a dual effect: on the one hand, the activation of microglia can promote the phagocytosis and clearance of Aβ; on the other, if the microglia is overactivated, they will induce the release of too many inflammatory mediators by the NF‐kB pathway, causing damage to synapses and nerves, leading to neuroinflammatory reactions (Figure 3). To prevent excessive activation, the homologous receptors expressed on microglia combine with a variety of neuronal immunomodulators to inhibit them, such as CD200/CD200R, CX3CL1/CX3CLR1, CD22/CD45, CD172A/CD47, and so forth.^7^ It is worth noting that high cholesterol and telomere shortening also play a role in the activation of microglia.^13^, ^18^ However, in drug research, Pls inhibits the endocytosis of TLR4 and the activation of downstream caspases, thereby inhibiting the activation of microglia.^19^ In a model expressing mutant human APP and mutant human progerin‐1 (PS1), it was found that minocycline also inhibited the activation of microglia.^20^

### 2.2 The mechanism of microglia in the body's aging

The occurrence of AD is closely related to aging. With age, the incidence of AD will gradually increase. Njie and his team, by isolating microglia from the central nervous system of old and young mice, found that the microglia of old mice not only constitutively secreted more TNF‐ α and IL‐6 but also induced a decrease in the level of glutathione and the ability to degrade Aβ. This shows that microglia are more easily activated in the brain of the elderly than young people.^21^ Some studies have found that the activity of microglia does not change during the process of aging, but only by enhancing the response to ifn‐γ to promote the activation of microglia and the transition to a pro‐inflammatory phenotype, leading to the disturbance of the inflammatory response mechanism.^11^ Furthermore, Olah et al. discovered a set of HuMi‐Aged genes preferentially expressed on microglia in the elderly and identified a special phenotype.^22^ In general, aging accelerates the activation of microglia and promotes the release of inflammatory cytokines from microglia.

### 2.3 Proliferation and senescence of microglia in AD; is it different from non‐AD?

Apart from he body's natural aging, is there any connection between the proliferation and aging of microglia itself and the pathogenesis of AD? Some studies have proved the connection between them. Bassani et al. used streptozotocin to evaluate the effect of neuroinflammation on adult neurogenesis and cognitive impairment in the STZ‐ICV rat model and found that the astrocytes and reactivity of the rat periventricular and hippocampus Glial cells increase considerably, and the proliferation of neural stem cells in the subventricular zone and the hippocampal dentate gyrus is significantly reduced.^23^ Moreover, some studies have observed that the colony‐stimulating factor 1 receptor expressed by microglia is an important receptor target for their proliferation, and IL‐34 secreted by neural stem cells can act on the colony‐stimulating factor 1 receptor to promote the proliferation of microglia and increase the clearance of soluble oligomeric amyloid‐β (oAβ), and have an adverse effect on the function of synapses and neurons in the brain of AD patients. However, the above finding was not observed in non‐AD^24^ patients (Table 1). In the study of Angelova and others, it can be seen that a large amount of iron accumulates in senescent microglia. Because iron (especially Fe(II)) can cause damage to intracellular macromolecules, when the iron is stored in a sufficient amount, a change in the phenotype of malnutrition can occur easily, which is manifested by the release of degraded ferritin and β‐amyloid. The protein factor is significantly less.^25^ Phosphorylation of senescent microglia significantly increases mammalian target of rapamycin (MTOR) levels while maintaining total protein levels unchanged. This explains the increase in endoplasmic reticulum pressure in aging microglia. Finally, acetylated NF‐kB (p65) increased, while total NF‐kB levels remained unchanged. This change is also related to reduced autophagy due to reduced SIRT‐1 activity, leading to an increase in endoplasmic reticulum stress and a corresponding decrease in autophagy, in turn resulting in a decrease in the insulin‐degrading enzyme (IDE). The reduction of IDE will lead to changes in ferritin expression and iron storage, affect the clearance of β‐amyloid protein, and reduce the degradation of the β‐amyloid protein^25^ (Table 2). In conclusion, the aging of microglia itself also promotes the occurrence of AD.

**Table 1:** Proliferation of microglia in AD and non‐AD patients

	Differences		Similarities
AD	1. Reactive microgliosis, astrogliosis, and an increase in TNF‐α levels	2. Il‐34 secreted by neural stem cells can promote clonal stimulator 1 receptor to promote microglial cell proliferation, increase clearance of soluble amyloid beta (oAβ), and impair synaptic and neuronal function.	After proliferation, microglia transform into macrophages to remove debris, and astrocytes proliferate to form glial scars, which play a role in repair and regeneration.
Non‐AD	Under normal circumstances, they do not proliferate, but interweave into a network, which plays a role in supporting neurons, biological metabolism, and nutrition. Microglia proliferate only when the center is damaged.		

Abbreviations: AD, Alzheimer's disease; TNF‐α, tumor necrosis factor α.

**Table 2:** Senescence of microglia in AD and non‐AD patients

AD/non‐AD	Differences	Similarities
AD	High levels of iron accumulate in aging microglia, and iron causes damage to intracellular macromolecules, resulting in significantly higher levels of phosphorylation in microglia, further affecting insulin secretion, and ultimately resulting in reduced clearance of beta‐amyloid.	With age, microglia senescence and intracellular ferritin will also increase.
Non‐AD	Microglia cells have less iron accumulation, more cell body branches and better synaptic sensitivit.	

Abbreviation: AD, Alzheimer's disease.

### 2.4 The influence mechanism of microglia on cognitive function

In AD, cognitive dysfunction is not related to Aβ or Tau pathology, but is related to hippocampal synaptic density. Increasingly more evidence has also shown that soluble ß oligomers combine with synapses to alter the shape, composition, and density of synapses, impairing cognition and long‐term enhancement. Neural stem cells (NSCs) may compensate for the toxic effects of oligomers on synaptic connections. NSCs can improve complex behavioral defects associated with a wide range of AD pathologies through brain‐derived neurotrophic factor (BDNF) (a bystander‐like mechanism).^26^ Baron et al.^27^ studied AD mouse models and found that the branching and protrusion of microglia decreased and the expression of pro‐inflammatory factors increased, resulting in a chronic pro‐inflammatory environment and aging. The ability of microglia to scan the environment is significantly reduced, so the microglia gradually shrink, leading to severe defects, and may cause a gradual decline in cognitive ability. In addition, studies by Bassani and his team have also proven that neuroinflammation and nerve damage can contribute to the occurrence of cognitive deficits.^23^ However, in the study of Felsky et al., from calculation of the AD genetic risk score, including and excluding the APOE effect, it was found that the overall risk of inflammatory diseases did not have such a strong impact on aging‐related cognitive decline, but it had no impact on peripheral immunity. Functional susceptibility variation has a greater impact, and at the same time, affects the number of microglia in the aging brain and regulates the expression of immune genes. It is necessary to further study the molecular mechanisms by which the risk of peripheral immune diseases affects glial cell activation to determine the key regulators of these pathways.^28^ Moreover, in late‐onset AD, a significant increase in cerebrospinal fluid progranulin (PGRN) is associated with severe cognitive impairment in AD patients. The increase in CSF PGRN is related to the increase in CSF soluble TREM2 (triggering receptor 2 on myeloid cells), but it may be related to the ability of sTREM2 to activate microglia.^9^ Most importantly, some receptor targets are also closely related to the occurrence of cognitive impairment. The lack or downregulation of the receptors SIRPα and CB1 on microglia and the receptor CD2000 on neurons will lead to microglia, and phagocytosis‐mediated increases in synapse loss and cognitive impairment.^29^, ^30^, ^31^ Additionally, blood‐derived fibrin acts on CD11b/CD18 of microglia, causing damage to synapses and also causing cognitive impairment.^32^

Belfiore, R. et al. evaluated female 3xTg‐AD and non‐transgenic (NonTg) mouse models and found that the Aβ load and Tau protein caused by Aβ deposition are located at Thr212/Ser214 or Ser202/Thr205 and Ser422. The phosphorylation of Tau protein leads to a neuro‐inflammatory response, which can also lead to a gradual decline in cognitive function.^33^ Ali et al.^19^ also found that spls reduces the expression of pro‐inflammatory cytokines by significantly reducing the endocytosis of TLR4 and inhibiting caspase‐3 activation, thereby increasing the activation of microglia to improve mild cognitive impairment. Most interestingly, Kaneshwaran et al.^34^ proved that as the degree of fragmented sleep increases, aging and activation of microglia also increase, which, to a certain extent, leads to the occurrence of cognitive impairment.

## 3 MICROGLIAL CELL‐MEDIATED AD TREATMENT PROGRESS

### 3.1 Receptor target therapy

Many studies have found that there are related receptor targets on microglia that can be used for AD treatment. For example, trigger receptor 2 (TREM2), NgR, and colony‐stimulating factor 1 receptor can all mediate the adhesion of microglia and migrate to Aβ deposition, absorption and removal of Aβ.^24^, ^35^, ^36^ In addition, Mildner et al. also proved that the chemokine receptor (CCR2) expressed on the surface of myeloid cells can also mediate microglia adhesion, migration, and clearance of Aβ.^37^ Specifically, myeloid cells expressing CCR2 were recruited as a preferred group to the deposition of amyloid beta (Aβ) to remove Aβ. Surprisingly, the brain of Alzheimer's disease patients lacking parenchymal bone marrow‐derived phagocytes and dysfunctional microglia showed no significant changes in plaque pathology and Aβ load. In contrast, the lack of CCR2 restricts perivascular myeloid cells significantly, reducing the clearance of β‐amyloid protein and increasing the deposition of Aβ in blood vessels, while the deposition of parenchymal plaques is not affected.^37^ Among the receptors expressed on the surface of microglia, the colony‐stimulating factor 1 receptor is activated by IL‐34, causing the proliferation of microglia and the elimination of soluble Aβ. IL‐34 increases the expression of insulin‐degrading enzyme and heme oxygenase‐1, aids in the elimination of oAβ, and reduces the neurotoxicity of soluble Aβ. Upregulation of colony‐stimulating factor 1 receptor expression can increase the effect of IL‐34 to improve cognitive impairment and reduce oAβ levels; NgR‐mediated Nogo inhibits microglia migration and fAβ (1‐42) adhesion, and the inhibitory effect of aging is also enhanced. Downregulation of NgR will promote the accumulation of microglia to Aβ deposits and increase the expression of CD36; upregulation of sTREM2 can reduce Aβ load, improve cognitive impairment, and enhance memory function. It is worth noting that the increased expression of sTREM2 can promote the migration of microglia and increase the clearance of Aβ, which has a neuroprotective effect.^24^, ^35^, ^36^ The above results all show that the four receptor targets of CCR2, colony‐stimulating factor 1 receptor, NgR, and sTREM2 can all be used as targets for the treatment of AD.

### 3.2 Drug (inhibitor) treatment and its mechanism of action

To date, many drugs have been studied and proven to prevent or reverse the pathology of AD disease by promoting the clearance of Aβ and Tau proteins and reducing the secretion of inflammatory factors. Although there has never been a complete cure for AD, AD drug research has entered the development stage.

#### 3.2.1 Promotion of Aβ clearance

Kummer et al. found that Myeloid‐related protein 14 (Mrp14) and its heterodimer Mrp8 were upregulated in microglia surrounding amyloid plaques. If Myeloid‐related protein 14 (Mrp14) is missing, it will lead to a decrease in key cytokines related to APP processing and Beta site amyloid precursor protein cleaving enzyme 1 (BACE1) downregulation, thereby increasing β deposition and promoting the phagocytosis of fibrous amyloid β (Aβ) by microglia.^38^ Il‐34 acted on the colony‐stimulating factor 1 receptor to increase the expression of insulin‐degrading enzyme and heme oxygenase‐1, thereby promoting the proliferation of microglia.^24^ Furthermore, blood protein fibrinogen inhibited the genetic ablation of CD11b or the generation of reactive oxygen species (ROS).^39^ Both of them facilitated the elimination of Aβ and reduced the neurotoxicity of soluble Aβ. In addition, some studies have proven that FLDK (an extract of Diospyros kaki leaves), WY peptides, hesperetin, MW‐151, Klotho, NaB, scyllo‐inositol therapy, and MRIgFUS/BAM10 + scyllo‐inositol combination therapy inhibit the excessive activation of microglia and promote the clearance of Aβ (Table 3).^40^, ^41^, ^42^, ^43^, ^44^, ^45^, ^46^

**Table 3:** Potential drugs for the treatment of AD and their mechanisms

Medicine	Mechanism	Results	References
IL‐34	Il‐34 acts on the colony‐stimulating factor 1 receptor and increases the expression of insulin‐degrading enzyme and heme oxygenase 1	Promotes Aβ clearance	Mizuno et al.^24^
Blood protein fibrinogen	Inhibits the genetic ablation of CD11b or the generation of reactive oxygen species (ROS)	Promotes Aβ clearance	Merlini et al.^39^
Mrp14	Mrp14 is missing, it will lead to a decrease in key cytokines related to APP processing, BACE1 downregulation, thereby increasing a β deposition	Promotes Aβ clearance	Kummer et al.^38^
FLDK	Inhibits the excessive activation of microglia	Promotes Aβ clearance	Ano et al.^40^
WY peptides	Inhibits the excessive activation of microglia	Promotes Aβ clearance	Bachstetter et al.^41^
Hesperetin	Inhibits the excessive activation of microglia	Promotes Aβ clearance	Ikram et al.^42^
MW‐151	Inhibits the excessive activation of microglia	Promotes Aβ clearance	Jiang et al.^43^
Klotho	Inhibits the excessive activation of microglia	Promotes Aβ clearance	Liu et al.^44^
NaB	Inhibits the excessive activation of microglia	Promotes Aβ clearance	Ma et al. (2018)
Scyllo‐inositol	Inhibits the excessive activation of microglia	Promotes Aβ clearance	Parthsarathy et al.^45^
MRIgFUS/BAM10 + scyllo‐inositol combination	Inhibits the excessive activation of microglia	Promotes Aβ clearance	Zeng et al.^46^
COX inhibitors and 5‐LOX inhibitors	Inhibit the toxic effects of microglia/macrophages in a dose‐dependent manner, inhibit the inflammatory cascade, and effectively reduce respiratory burst activity	Reduce inflammatory response	Klegeris and McGeer^47^
Eriodictyol	Inhibits acetylcholine, and increases ChAT activity and ACh level. Inhibits the excessive activation of glial cells, and inhibits the MAPK and NF‐κB pathways	Reduces inflammatory response	Ilievski et al.^48^
SPls	Inhibit lipopolysaccharide‐mediated TLR4 endocytosis, and block the activation of caspases	Reduce inflammatory response	Ali et al.^19^
Mogrol	Inhibits excessive activation of cells and activation of NF‐κB signaling	Reduces inflammatory response	Chen et al.^49^
MpHE	Inhibits astrogliosis, promotes microglia phagocytosis, and inhibits the pro‐inflammatory M1 phenotype of microglia	Reduces inflammatory response	Medrano‐Jiménez et al.^50^
JNJ‐527	inhibits CSF1R phosphorylation and reduces the expression of Il‐1b and TNFa	Improves tau pathology	Mancuso et al.^51^
OAB‐14	promotes microglial phagocytosis and increases the expression of IDE and NEP	Improves tau pathology	Yuan et al.^52^

Abbreviations: 5‐LOX, 5‐lipoxygenase; AD, Alzheimer's disease; COX, cyclooxygenases; FLDK, flavonoids extracted from leaves of Diospyros kaki; IL‐34, interleukin‐34; Mrp14, myeloid‐related protein 14; SPlS, SQUAMOSA PROMOTER BINDING PROTEIN‐LIKE.

#### 3.2.2 Reduction of inflammatory response

Some studies have found that many drugs play a significant role in the treatment of AD by inhibiting the inflammatory response. Ilievski^48^ and his team found that eriodictyol reduced the deposition of Aβ, and that high‐dose eriodictyol inhibited acetylcholine, and increased ChAT activity and ACh level to balance the cholinergic system. Eriodictyol can also inhibit the excessive activation of glial cells, and reduce the secretion of inflammatory factors and inflammatory mediators by inhibiting the MAPK and NF‐κB pathways, and reducing nerve damage and amyloidosis. Interesting, sPls, Mogrol, and the hydroethanolic extract of thrips leaf (MpHE) all have the effect of inhibit neuroinflammation, which was found by Chen and Medrano‐Jiménez et al. The specific mechanisms are as follows: sPls significantly inhibited lipopolysaccharide‐mediated TLR4 endocytosis and blocked the activation of caspases^19^; Mogrol can inhibit the microglia activation excessive activation of cells and activation of NF‐κB signaling caused by Aβ (1‐42)^49^; and MPHE promotes microglia phagocytosis and inhibits the pro‐inflammatory M1 phenotype of microglia.^50^ Finally, a reduction of pro‐inflammatory factors occurs, thereby improve mild cognitive impairment. Neurobiol et al. cultured neuron‐like SH‐SY5Y cells in the supernatant of activated cells of the THP‐1 line of human monocytes and observed them. Respiratory bursts were observed and possible neuroprotective drugs were tested. It is found that both COX inhibitors and 5‐LOX inhibitors may inhibit the toxic effects of microglia/macrophages in a dose‐dependent manner, inhibit the inflammatory cascade, and effectively reduce the respiratory burst activity. COX inhibitors include indomethacin, NS‐398, ibuprofen, and nitric oxide derivatives of indomethacin, ibuprofen, and flurbiprofen; 5‐LOX inhibitors include REV 5901 and 5‐LOX Activating protein (FLAP) inhibitor MK‐886, and the combination of the two may have greater therapeutic potential than a single inhibitor (Table 3).^47^

#### 3.2.3 Improvement of Tau pathology

Mancuso et al. identified JNJ‐40346527 (JNJ‐527), a CSF1R inhibitor that inhibits microglia proliferation by inhibiting CSF1R phosphorylation, resulting in decreased expression of genes associated with microglia proliferation. Meanwhile, JNJ‐527 can also reduce the expression of Il‐1b and TNFa, reduce inflammation, play a neuroprotective role, and induce improvements in cognitive impairment.^51^ OAB‐14 promotes microglial phagocytosis and increases the expression of IDE and NEP to promote Aβ clearance. Because of the clearance of Aβ, synaptic degeneration and Tau protein hyperphosphorylation were reduced, which led to a decrease in neuron loss and neuroinflammation (Table 3).^52^ The above research results prove that the above‐mentioned drugs and cytokines can be used to effectively treat AD by improving the pathology of Tau.

### 3.3 Other treatments

Over the recent years, Liang, F. and others have studied the effects of social interaction on the AD pathology and cognitive function of APP/PS1 mice, and found that mice with high‐quality social interactions have significantly reduced plaques produced by amyloidosis. The related anti‐inflammatory genes in microglia are significantly upregulated, which enhances the anti‐inflammatory effect and neuroprotective function. This indicates the importance of social activities in the prognosis of AD patients.^53^ Interestingly, Wang et al. used AD mouse models and found that changes in intestinal flora can cause isoleucine and phenylalanine to accumulate in peripheral blood, leading to pro‐inflammatory T helper cell proliferation and differentiation. This leads to activation of M1 microglia, causing inflammation and cognitive impairment. This shows that the imbalance of the intestinal flora in AD patients will promote the inflammatory response, and a new strategy for the treatment of AD is proposed: reshaping of the intestinal microbiota.^54^

## 4 CONCLUSION

In this review, I described the role of microglia in AD pathology and found that the activation of microglia induces the release of related cytokines to participate in the subsequent neuroinflammatory response, which has an impact on cognitive function. However, pathological reactions such as neuroinflammation and cognitive impairment occur mainly due to the accumulation of β‐amyloid protein in the microglia and the phosphorylation of Tau‐like protein, resulting in the formation of neurofibrillary tangles. This review also focused on the treatment of microglia‐mediated AD, which can be roughly divided into three aspects: receptor target therapy, drug therapy, and other therapies. I found that most of the studies focus on promoting the clearance of Aβ to alleviate the pathological state, and there is almost no research on alleviation of the pathological phenomenon by alleviating the Tau‐like protein phosphorylation pathway. Perhaps, this is because Tau‐like protein exists in the cell and has the effect of maintaining the stability of microtubules, so it is not easy to handle. I believe it can inhibit the effect of phosphokinase, leading to phosphorylation of Tau‐like proteins and thus prevent phosphorylation of Tau‐like proteins. This may also be a way to treat AD, and it may represent a new avenue for the treatment of AD in the future. Hopefully, one day, we will find a complete cure for this disease.

## CONFLICT OF INTERESTS

The authors declare that there are no conflicts of interest.

## ETHICS STATEMENT

The ethics statement is not available.

## AUTHOR CONTRIBUTIONS

Yi‐Huan Guan, Ling‐Jing Zhang, Shi‐Ya Wang, and Ya‐Dan Deng completed the manuscript. Dong‐Qing Chen, Lan‐Chun Zhang, and Hong‐Su Zhou polished the whole article. Lan‐Chun Zhang finalized and approved this paper. All authors have read and approved the final submitted manuscript.

## TRANSPARENCY STATEMENT

The authors affirm that this manuscript is an honest, accurate, and transparent account of the study being reported; that no important aspects of the study have been omitted; and that any discrepancies from the study as planned (and, if relevant, registered) have been explained.

5

## Data Availability

Data sharing not applicable to this article as no datasets were generated or analysed during the current study.
